# Draft Genome Sequence of Fusarium oxysporum f. sp. *cubense* Tropical Race 4 from Peru, Obtained by Nanopore and Illumina Hybrid Assembly

**DOI:** 10.1128/mra.00347-22

**Published:** 2022-08-08

**Authors:** Ana M. Leiva, Mathieu Rouard, Diana Lopez-Alvarez, Alberto Cenci, Catherine Breton, Rosalyn Acuña, Juan Carlos Rojas, Miguel Dita, Wilmer J. Cuellar

**Affiliations:** a Crops for Nutrition and Health, International Center for Tropical Agriculture (CIAT), Palmira, Colombia; b Bioversity International, Montpellier, France; c Facultad de Ciencias Agropecuarias, Universidad Nacional de Colombia, Palmira, Colombia; d Servicio Nacional de Sanidad Agraria (SENASA), Lima, Peru; e Instituto Nacional de Innovación Agraria (INIA), Lima, Peru; f Bioversity International, Cali, Colombia; Vanderbilt University

## Abstract

Fusarium oxysporum f. sp. *cubense* tropical race 4 (Foc TR4) is the causal agent of Fusarium wilt, a major threat to the banana industry worldwide. Here, we report the genome of a Foc TR4 strain from Peru, sequenced using a combination of Illumina and Oxford Nanopore Technologies.

## ANNOUNCEMENT

Fusarium wilt of banana, caused by Fusarium oxysporum f. sp*. cubense*, is a devastating fungal disease affecting bananas worldwide. The pathogen population is divided into four races, but tropical race 4 (Foc TR4) is by far the most aggressive, as it attacks several banana types, including Cavendish, which dominates the global banana export economy (1). In the last 6 years, Foc TR4 has spread from Asia into the Middle East and Africa (2), and in 2019, Foc TR4 reached Latin America, in the north of Colombia (3).

In April 2021, banana plants (Musa acuminata group AAA, subgroup Cavendish) showing symptoms of Fusarium wilt were observed on a farm in Querecotillo, Peru (4°43′54.84″S, 80°33′45.00″W). Diagnostic analyses confirmed the identity of the pathogen as Foc TR4 (4). Pseudostem strands from symptomatic plants were transferred to potato dextrose agar (PDA) medium and incubated at 25°C. Single-spore isolates from fungal colonies identified as Fusarium oxysporum species complex were further purified and used for DNA extraction (5). DNA from 4 samples (PerS1 to PerS4) was extracted using the Illumina DNA prep kit and sequenced using the MiSeq platform (2 × 151 bp). The same DNA sample from PerS4 was further used for sequencing with Oxford Nanopore Technology (FLOW-MIN111, R10.3 chemistry, LSK109 kit) (6). A total of 46,707,802 Illumina and 379,956 Nanopore reads (average length, 2,783 bp) were obtained. The filtered reads (Illumina, 93.60% > Q30) were combined using Unicycler v0.4.8 (7) to make a hybrid genome assembly with a total length of 46,361,425 bp distributed in 115 contigs (G+C content, 47.59%; *N*_50_, 1.63 Mbp). The consensus sequence quality, checked using Qualimap v2.2.1 (8), resulted in an average depth of 19.25× with 6,718,534 reads mapped.

**FIG 1:**
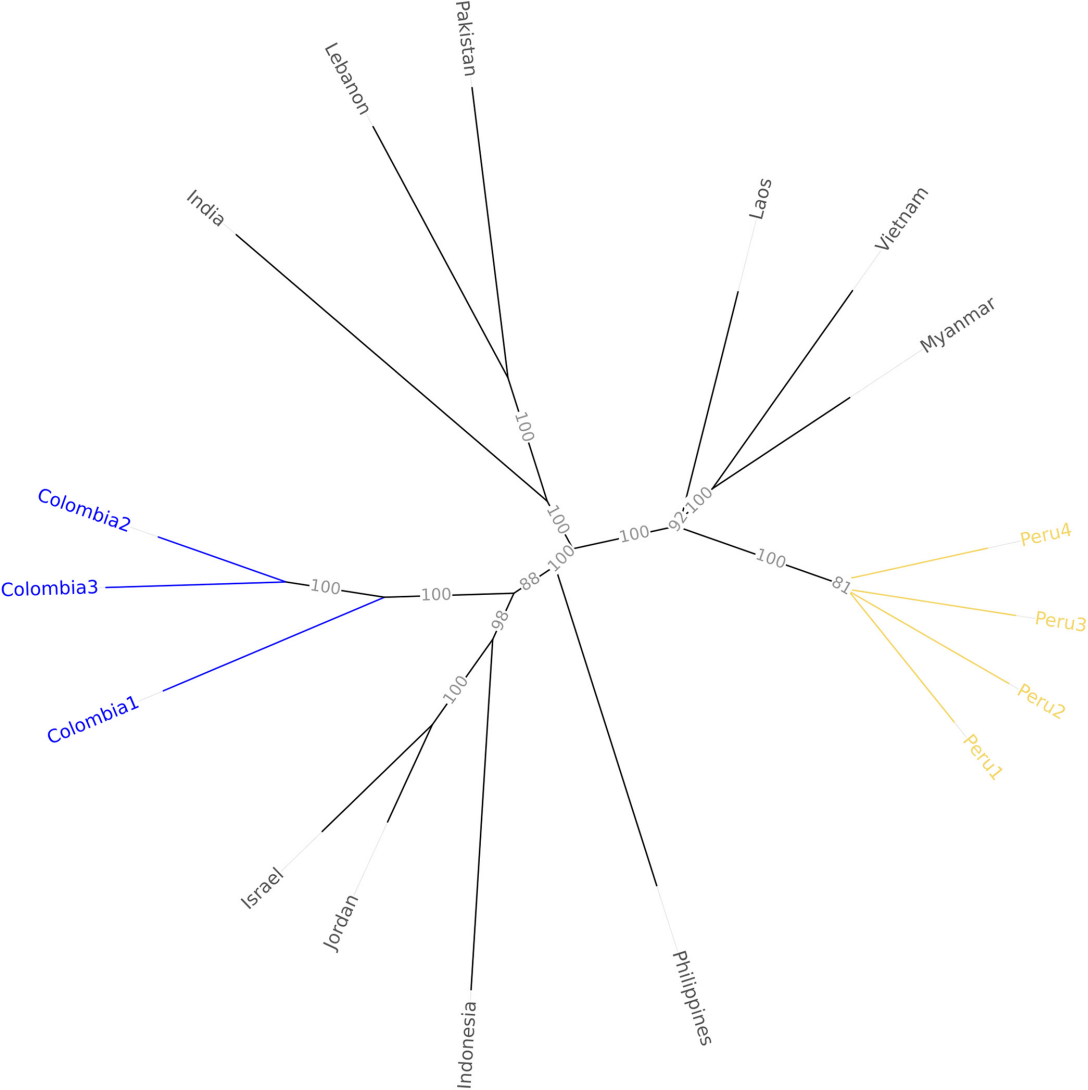
Neighbor-joining phylogenetic tree of 17 Fusarium oxysporum f. sp. *cubense* TR4 isolates with 6,462 SNP variants. Support bootstrap values are indicated as a percentage of the 1,000 replicates. The sequences included were from Colombia (SRA accession number SRR10747097, SRR10125423, SRR10103605), India (SRR13311628), Indonesia (SRR10054446), Israel (SRR10054450), Jordan (SRR10054448), Laos (SRR7226878), Lebanon (SRR7226880), Pakistan (SRR7226883), Peru (SRR15514269 to SRR15514272), and the Philippines (SRR10054447) and were mapped onto strain UK0001 (GenBank accession number GCA_007994515.1).

The sequence assembly, mapped using QUAST v5.0.2 (9), showed high contiguity and a total aligned length of 45.9 Mb (94.7% genome fraction) with the highest-quality genome sequence available, strain UK0001 (10). Gene space assessment was performed using BUSCO v5.2.2 (11) (hypocreales odb10), which reported 97.7% completeness (single copy, 97.2%; duplicate, 0.5%; fragmented, 0.5%; missing, 1.8%; *n* = 4,494). The hybrid assembly was used to identify a family of *s*ecreted *i*n *x*ylem (SIX) genes. The presence or absence of the SIX homologs was checked using BLASTN v2.2.26 (Table 1) to identify the F. oxysporum f. sp*. cubense* sequences and matched the expected allelic variants identified in Foc TR4 for SIX1, SIX6, and SIX8 (12). Moreover, the PerS4 reads, combined with previously reported Foc TR4 strains (2, 3, 13–15), were mapped on UK0001 (10) using BWA v0.7.15 (16), and single nucleotide polymorphism (SNP) calling was conducted using GATK v4.1.6 (17). A dissimilarity matrix (simple matching index) and a neighbor-joining phylogenetic tree were subsequently computed using Darwin v6 (18) (Fig. 1). Peruvian samples were clustered together, separated from other strains, including those from Colombia, suggesting independent incursions of Foc TR4 in the Americas (Fig. 1). Default parameters were used for all software unless otherwise specified.

**TABLE 1:** Representative subset of SIX gene homologs detected in isolates of Fusarium oxysporum f. sp. *cubense*^*a*^

Race	VCG^*b*^	BRIP accession code^*c*^	Presence of SIX gene:							
			1	2	4	6	7	8	9	13
R1	0123	62895	x^d,f^		x^b^	x^b^			x^a^	x^a^
R2	01214	25609	x^f^						x^a,c^	x^a^
R4	0122	62892	x^c,i^					x^a3^	x^a^	x^c^
STR4	0120	44012	x^g^	x^d^	x^a^		x^a^	x^a3,b^	x^a^	
TR4	01213	40340	x^a,h,i^	x^a^	x^c^	x^a^		x^a1,a2^	x^a^	x^a,e^
TR4^*d*^			x^a,h,i^	x^a^	x^c^	x^a^		x^a1^	x^a^	x^a,e^

a Isolates shown were reported in reference 12, with the addition of the TR4 Peruvian allelic variants. Sequences were searched on the assembly using BLAST matching with high similarity. X denotes the presence of a gene, while the superscript letters correspond to allelic variants of the gene (TR4 SIX1a, h, i: GenBank accession numbers KX434991, KX434998, KX434999, respectively; SIX2a: KX435000; SIX4c: KX435006; SIX6a: SIX8a1: KX435011, KX435012; SIX9a: KX435015; SIX13: KX435019, KX435023). SIX, secreted in xylem.

b VCG, vegetative compatibility group.

c BRIP, Queensland Plant Pathology Herbarium.

d Peruvian samples.

The availability of complete genome sequences and their comparative analysis will contribute to a better understanding of the Foc TR4 population biology, disease epidemiology, and management.

### Data availability.

The sequence reads have been deposited at GenBank under the BioProject accession number PRJNA755905, and the assembly sequence has been deposited under the GenBank accession number GCA_021237285.1.
